# Ethical and clinical challenges in managing low-penetrance CNVs: insights from Portuguese clinical geneticists

**DOI:** 10.3389/fgene.2026.1757167

**Published:** 2026-03-02

**Authors:** Ana Rita Soares, Jorge Diogo Da Silva, Alberto Caldas Afonso, Ana Sofia Carvalho

**Affiliations:** 1 Clinical Genetics Department, Clínica de Genética e Patologia, Unidade Local de Saúde de Santo António, Porto, Portugal; 2 UMIB–ICBAS, UP / Unit for Multidisciplinary Research in Biomedicine / Instituto de Ciências Biomédicas Abel Salazar, Porto University, Porto, Portugal; 3 Life and Health Sciences Research Institute (ICVS), School of Medicine, University of Minho, Braga, Portugal; 4 ICVS/3B’s – PT Government Associate Laboratory, Braga, Portugal; 5 Centro Materno-Infantil do Norte, Unidade Local de Saúde de Santo António, Porto, Portugal; 6 ICBAS-UP – Instituto de Ciências Biomédicas Abel Salazar, Porto University, Porto, Portugal

**Keywords:** ethics in genomic medicine, genetic counselling, low-penetrance copy number variants, neurodevelopmental disorders, reproductive decision making

## Abstract

**Introduction:**

Low-Penetrance Copy Number Variants (LP-CNVs) are well-known to contribute to neurodevelopmental disorders and are also found in healthy individuals, presenting significant challenges to genetic counselling. However, data on the clinical management of LP-CNVs in Portugal is lacking.

**Methods:**

An online questionnaire was administered to Clinical Geneticists in Portugal regarding their management of LP-CNV and ethical issues were addressed.

**Results:**

The results showed a significant absence of agreement on LP-CNVs disclosure, particularly concerning whether decisions should be guided by expert panels or individualized for each case. Clinicians acknowledged the substantial challenges patients and families face in understanding genetic information, highlighting the need for a shared decision-making approach. Furthermore, there was considerable variability in ethical perspectives regarding prenatal diagnosis and preimplantation genetic testing for LP-CNVs, emphasizing the need for clear guidelines. Our results strongly advocate for the development of national guidelines mirroring those established in other countries.

**Discussion:**

This work underscores several complex ethical issues requiring urgent exploration internationally. The observed postcode lottery highlights a failure of distributive justice, necessitating equitable access to standardized genomic knowledge across healthcare regions. Furthermore, the uncertainty challenge renders traditional non-directive counseling increasingly unsustainable, requiring a shift toward Shared Decision-Making (SDM) to balance child welfare against parental autonomy in navigating genomic ambiguity.

## 1 Introduction

Copy Number Variants (hereafter CNVs) are well-known causes for several genetic syndromes and have been associated with multiple pathologies, from pediatric (such as congenital anomalies, global developmental delay (DD) and/or intellectual disorder (ID), autism spectrum disorder (ASD) and epilepsy) to adulthood (such as schizophrenia and psychiatric pathology), and from asymptomatic to very severe (Rosenfeld et al., 2013; Morris et al., 2021; Miller et al., 2010). Due to the increasing availability of array techniques in the clinical setting, CNV detection is at an all-time high.

Low-penetrance CNVs (LP-CNVs) are deletions or duplications found in a small percentage of patients with clinical manifestations and a higher percentage of the healthy population. Although a cut-off of 20% for penetrance can be used to consider a CNV as low-penetrant, it is interesting to notice the wide variation in penetrance for different CNVs, with different clinical implications (Rosenfeld et al., 2013; Goh et al., 2025; Cooper et al., 2011). This ambiguity in their clinical relevance creates substantial downstream dilemmas for clinical geneticists regarding classification, reporting, and genetic counselling, and this has been a subject of debate in the medical genetics community. While some groups classified these CNVs as pathogenic variants of low penetrance, others classified them as susceptibility loci for NDD, and others considered them variants of high penetrance with variable expressivity (Kendall et al., 2019; Muys et al., 2020; Govaerts et al., 2017; Shkedi-Rafid et al., 2016). More recently, some research groups concluded that “healthy” CNV carriers may have better performance than affected cases, but have lower education levels and less differentiated professions compared to non-carriers. Kendall et al. also analyzed a population carrying these CNVs described as “normal”, and concluded that these individuals have subtle cognitive deficits, notably at the educational/school level and in their ability to earn a salary (Kendall et al., 2019).

These LP-CNVs are a frequent cause for referral patients to Clinical Genetics services: a report from Newcastle upon Tyne in 2019 revealed that, in 3 years, 163 cases were observed in the genetics clinic (including only the top 10 most common LP-CNVs), showing that a Clinical Geneticist observes at least 54 cases of LP-CNVs per year (National Library of Medicine, 2020) L. Govaerts et al. found these alterations in 1%–3% of pregnancies tested prenatally, depending on the cohort selection (Govaerts et al., 2017).

Furthermore, LP-CNVs can be detected in different scenarios: i) as the genetic etiology, both in pre or postnatal settings, where the CNV explains a phenotype; ii) as an incidental finding, both in pre or postnatal settings, when the detected CNV does not explain the phenotype; iii) in the study of familial CNV in so-called “normal and healthy” parents/family members. Interestingly, in the Newcastle upon Tyne study, CNVs were considered the cause for clinical manifestations in 78.5% of cases. In about 16.6% of cases, additional genetic testing was offered (and the CNV was considered a possible incidental finding) (Christiansen et al., 2004).

As such, LP-CNVs pose daily clinical and ethical challenges for Clinical Geneticists, particularly concerning disclosure (especially in the prenatal setting where LP-CNVs may be incidental findings) and the discussion of familial testing and reproductive options (mainly prenatal diagnosis (PND) or preimplantation genetic testing (PGT)).

Accordingly, countries such as the United Kingdom and Belgium have established guidelines on which LP-CNVs should be reported, how they should be classified and addressed (depending on the clinical scenario), and what the repercussions of these kinds of results (particularly in familial testing and reproductive options) (Gardiner, 2015) (BeSHG, 2024). As far as we know, there is currently no national guidance governing the interpretation or clinical management of LP-CNVs in most countries, including in Portugal.

To address this gap, we conducted a nationwide survey of Portuguese clinical geneticists to document current practices and professional attitudes regarding the classification, reporting, and ethical management of LP-CNVs. The study aims to characterize inconsistencies in clinical decision-making, identify areas of greatest professional uncertainty, and use these insights to inform the development of a targeted, evidence-based framework for future national guidelines, with implication beyond Portuguese context. Moreover, we propose to undertake a more in-depth analysis of the ethical issues inherent in current LP-CNV management.

## 2 Methods

### 2.1 Study population, data collection and study type

A survey regarding the personal experience of Clinical Geneticists in the diagnosis and management of LP-CNVs was conducted. This questionnaire was partially based on Shkedi-Rafid, S. questionnaire (Shkedi-Rafid et al., 2016) (translated to Portuguese), and it was composed of two sections: section one, related to demographic data; and section two, related to the state of LP-CNV management (S1 - Supplementary Questionnaire). Section two comprised 32 questions on a 5-point Likert scale (1 for “strongly disagree”, 2 for “disagree”, 3 for “neither agree nor disagree”, 4 for “agree” and 5 for “strongly agree”).

The study population comprised all 43 Medical Genetics specialist physicians with active practice in the Portuguese National Health System by the end of 2022. In Portugal, the profession of genetic counsellor is not officially recognized, due to a combination of historical, legal, and structural factors within the healthcare system. As a result, the genetic counselling process is restricted to medical geneticists. All physicians were directly contacted via email with the survey in an attachment, and at least three contact attempts were made. The survey was electronically available in Google Forms (Google Forms Google LLC, California, EUA), and responders could directly submit their answers through this platform. The first contact was performed on 26th November 2022, and the survey was open for response until 1st March 2023. The questionnaire was fully anonymized.

The study was observational and cross-sectional, solely comprising descriptive and inferential analysis of data obtained from the survey responses.

### 2.2 Statistical analysis

Categorical variables were represented by their frequency and relative proportion; ordinal variables were represented by the median and interquartile range (IQR). In all statistical tests, the assumed confidence level was 95%. A Fisher’s exact test was performed to compare proportions between the two groups, and Cramer’s V was used as an effect size measure. Cutoffs for Cramer’s V magnitude were 0.100, 0.300 and 0.500 for small, medium and large effects, respectively. Kendall’s tau-b correlation test (two-sided) was employed to assess for correlations between two ordinal variables. 95% confidence intervals for Kendall’s tau-b were obtained after applying Fisher’s z-transformation. Cutoffs for Kendall’s tau-b magnitude were 0.100, 0.300 and 0.500 for small, medium and large effects, respectively. There was no missing data in any of the correlation assessments. Multiple hypothesis testing adjustment (such as with the Bonferroni correction) was not performed due to the exploratory nature of this study and the population’s small sample size, in order to avoid inflation of type II error/excessive reduction of statistical power.

Dimension-reduction analyses for the second section of the survey were not possible due to sample size limitations, and therefore, formal survey validation was not possible. Predictively, a sample size of about 10–15 times the number of measured variables would be suitable (in this case, about 320–480 participants); otherwise, a minimum number of 300 participants has also been reported as a sufficient sample size for a quality factor analysis (MacCallum et al., 1999). In our case, the complete population comprises 43 subjects, and it would therefore be impossible to meet the sample size requirements. Selecting a maximum of 4-5 variables for assessment would also be insufficient to generate factor data. Finally, the correlation matrix of our variables is not positive definite. Therefore, the Kaiser-Meyer-Olkin statistic cannot be computed, which also indicates the lack of suitability of our sample size. For these reasons, we opted to perform direct correlation analyses only as a measure of survey validation. All tests were performed using SPSS® version 26.0 or GraphPad Prism® version 9.3.0.

### 2.3 Ethical statement

Before the survey, respondents had to return informed consent for this study, with all relevant information displayed in the first section of the form. The study was approved by the center’s Ethics Committee (Protocol TA-DT, Ref. 2022-146(118-DEFI/120-CE)).

## 3 Results and discussion

### 3.1 Demographic characterization of the sample

By December 2022, a total of 43 Clinical Geneticists worked on national healthcare services, either full or part-time (17 in the north, 10 in the center, and 16 in the south–according to the Nomenclature of Territorial Units for Statistics (NUTS)). Of 43 individuals, 24 (56%) responded to the anonymized online questionnaire, which we considered sufficiently representative to give us an overview of the Portuguese reality regarding LP-CNVs management. Table 1 shows relevant demographic data, as well as which genetic tests are prescribed by the medical doctor and to where (private or public laboratory). Regarding geographical distribution, 46% (n = 11) of respondents were from the North, 33% (n = 8) from the South, and 21% (n = 5) from the Center of Portugal. In terms of professional experience, 58% (n = 14) had been medical genetics specialists for more than 5 years, while 42% (n = 10) had 5 years or less. Regarding diagnostic infrastructure, 46% (n = 11) utilized internal laboratories, while 42% (n = 10) relied on external private laboratories. The most commonly prescribed tests for CNV detection were CGH-Array (96%) and MLPA (92%). Some demographic data (such as sex or working hospital) was not collected in order to mitigate the risk of re-identification.

**TABLE 1:** General characterization of the sample. CGH, comparative genomic hybridization; MLPA, multiplex ligation-dependent probe amplification; NGS, next-generation sequencing; SNP, single nucleotide polymorphism. N (%), frequency (proportion).

Variable		N (%)
Geographical region of work	North	11 (46%)
	Center	5 (21%)
	South	8 (33%)
Years as medical genetics specialist	≤5	10 (42%)
	>5	14 (58%)
Laboratory performing tests	Internal	11 (46%)
	External – Public	3 (13%)
	External – Private	10 (42%)
Commonly prescribed tests for CNV detection	CGH-array	23 (96%)
	SNP-array	12 (50%)
	MLPA	22 (92%)
	NGS	2 (8%)

In Portugal, clinical genetics services are almost exclusively based within the public National Health Service, and most centers rely on external laboratories for genetic testing rather than performing these analyses in-house within public hospitals. As these laboratories operate as commercial entities, case discussions and decision-making processes can at times be challenging.

Regarding common practices in the management of NDD cases with CNVs from the responding Clinical Geneticists (Table 2), we observed that the majority of physicians discusses prenatal cases with the Fetal Medicine team and refers postnatal cases to Developmental Pediatrics or Child Psychiatry, while involving primary care is less frequent. Most physicians also reevaluate cases as requested an in the pre-conception setting.

**TABLE 2:** Responses regarding different management strategies employed by each Clinical Geneticists for NDD patients that are CNV carriers. CNV, copy-number variant. NDD, neurodevelopmental disorder.

Management strategies for CNVs in NDD	Positive responses
Discuss cases with the fetal medicine team	18 (75%)
Refer patients to developmental pediatrics or child psychiatry	21 (88%)
Refer patients to primary care physician	5 (21%)
Reevaluate cases at the time of reproductive planning	19 (79%)
Reevaluate cases when requested by other physicians	18 (75%)

### 3.2 Overall responses to the survey

**FIGURE 1:**
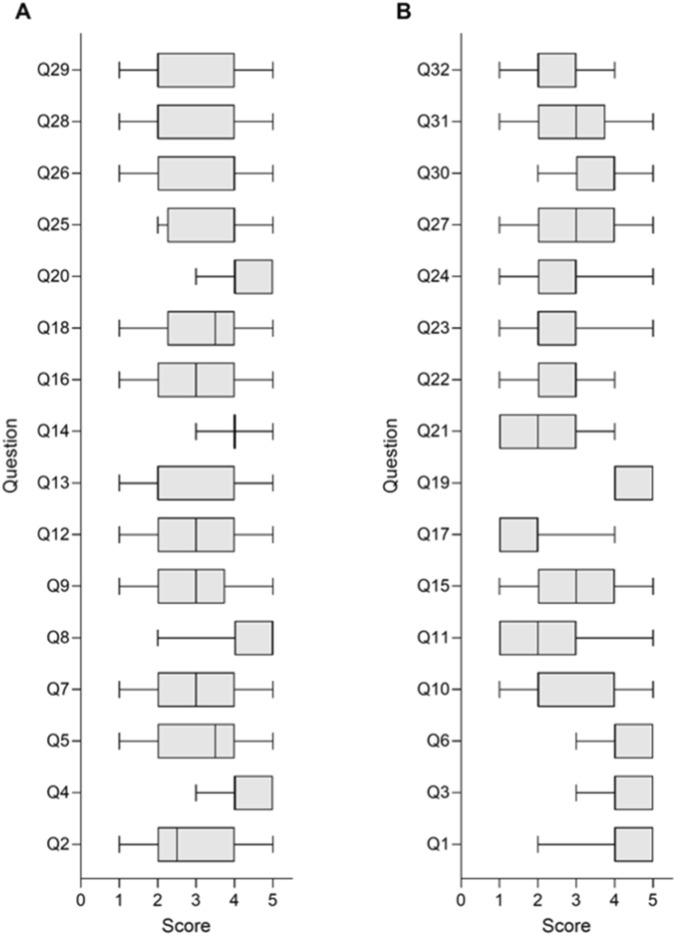
Box plot graphic titled “A” and “B” side by side, displaying the distribution of scores for survey questions regarding the assessment of who decides upon disclosure and when does it happen **(A)**; and regarding the impact of disclosure in patients, families and embryos/fetuses **(B)**. Each box plot visualizes median, interquartile range, and variability of scores from zero to five for individual questions.

Figure 1 presents the results of the survey’s Likert type questions (median and interquartile range of response scores), per individual question, regarding the assessment of who decides upon disclosure and when does it happen (Figure 1A); and regarding the impact of disclosure in patients, families and embryos/fetuses (Figure 1B). Regarding answer distribution, we have already observed widespread responses in several questions by visual inspection. These results were obtained to assess different aspects of the responders’ approaches and views on LP-CNV classification and management. They will be used to assess for correlations between these approaches/views and as a measure of survey validation.

### 3.3 Current reporting practices

We started by assessing which variant types, per the ACMG classification criteria, into which LP-CNVs are classified, either in the prenatal or postnatal setting (Table 3). It is interesting to highlight that different Portuguese laboratories report LP-CNVs as all possible classification subtypes ((likely) benign, VUS and (likely) pathogenic), while these variants are known to be (likely) pathogenic with low-penetrance. Moreover, the choice of classification did not differ when comparing LP-CNVs in the prenatal and postnatal settings. These results show a high rate of ambiguity and lack of consensus on this matter.

**TABLE 3:** Types of variant classification reported for low-penetrance CNVs in the prenatal and postnatal settings, according to the laboratory reports medical doctors deliver to patients. N (%), frequency (proportion). It is important to notice that variants of unknown significance lack sufficient evidence for interpretation, whereas variants of uncertain significance have conflicting or incomplete clinical data that prevent definitive classification, representing different levels of ambiguity.

Type of variant	Prenatal, N (%)	Postnatal, N (%)	χ^2^	Sig	Cramer’s V
Pathogenic/likely pathogenic	22 (92%)	23 (96%)	0.356	0.999	0.086
Variant of unknown significance	6 (25%)	9 (38%)	0.873	0.534	0.135
Variant of uncertain significance	8 (33%)	11 (46%)	0.784	0.556	0.128
Variant of uncertain or unknown significance (not discriminated)	11 (46%)	13 (54%)	0.333	0.773	0.083
Variant of susceptibility	3 (13%)	5 (21%)	0.600	0.701	0.112
Benign/likely benign	12 (50%)	15 (63%)	0.762	0.561	0.126

This situation poses several clinical and ethical issues, mainly due to the outcome of these results, which we propose to analyze through different clinical scenarios:

• In the first scenario, the lab reports the CNV as (likely) pathogenic and genetic counselling is performed as such, with reproductive options being offered to the couple and follow-up oriented accordingly.
• In the second scenario, the lab reports the CNV as benign, a situation that can be criticized, as according to ACMG guidelines, benign variants must not be reported in any clinical situation.
• In the third scenario of reporting CNV as a VUS (or even susceptibility locus), issues such as what to do with the result, what to tell patients or parents, and which reproductive options should be offered can be posed.

### 3.4 Validation of survey responses

The correlation matrix between the scores of each survey question is presented in Supplementary Table S2 (S2). Although dimension-reducing tests were not employed, and therefore we were not able to extract specific factors, we were able to group specific questions in general topics regarding LP-CNV management (Table 4): i) Disclosure Decisions; ii) Impact on Patients/Families; iii) Reproductive. In Table 4, each question of the survey was categorized à posteriori into one of the specific themes adapted from the 4W’s tool (the four basic questions: Who? What? Where? Why?) to gain a clearer picture of disclosing LP-CNVs by Clinical Geneticist in Portugal: i) Who decides to disclose; ii) When to disclose; iii) How disclosure impacts patients/families; iv; How disclosure impacts fetus/embryos.

**TABLE 4:** Grouped questions by main theme and specific theme. “General” means that the question has more than one statistical correlation; “X” means that the question has no strong or moderate correlation.

Question number	Overall theme	Specific theme	Question
2	General	When to disclose	The laboratory should report all findings, regardless of their clinical significance, only in postnatal cases
6	General	How disclosure impacts patients/families	Disclosure incidental findings can lead to parental anxiety
7	General	Who decides to disclose	I Believe the clinician should “choose” the findings to disclosure based on what the parents want to know
8	General	Who decides to disclose	The decision on which information to disclose, especially regarding variants of uncertain clinical significance, should be determined by national guidelines and not left to individual laboratories/clinicians
15	Reproductive options	How disclosure impacts fetus/embryos	When a low penetrance variant explains the proband’s phenotype, clinicians should offer invasive prenatal diagnosis in future pregnancies
16	Reproductive options	When to disclose	The laboratory should disclose only variants that provide a clinical explanation for the found fetal anomaly
22	Reproductive options	How disclosure impacts fetus/embryos	Embryos carrying low penetrance CNVs should be “excluded”
24	Reproductive options	How disclosure impacts fetus/embryos	In my service, when a low penetrance variant explains the proband’s phenotype, clinicians offer pre-implantation genetic diagnosis
27	Reproductive options	How disclosure impacts fetus/embryos	In my service, when a low penetrance variant explains the proband’s phenotype, clinicians offer invasive prenatal diagnosis in future pregnancies
28	Reproductive options	When to disclose	The laboratory should disclose all findings, regardless of their clinical significance, even in prenatal cases
31	Reproductive options	How disclosure impacts fetus/embryos	When a low penetrance variant explains the proband’s phenotype, clinicians should not offer pre-implantation genetic diagnosis
3	Impact on patients/Families	How disclosure impacts patients/families	I Consider that parents/probands may have difficulty understanding the information provided in genetic counseling, especially the possible findings of the genetic study
14	Impact on patients/Families	When to disclose	In my opinion, variants of uncertain clinical significance/low penetrance should be reported
19	Impact on patients/Families	How disclosure impacts patients/families	I Consider that parents/probands may have difficulties understanding the information provided in genetic counseling
20	Impact on patients/Families	When to disclose	In my service, variants of uncertain clinical significance/low penetrance are disclosed
21	Impact on patients/Families	Who decides to disclose	I Believe the clinician receiving the report should decide what information to give to parents/probands
23	Impact on patients/Families	How disclosure impacts patients/families	In my service, when a low penetrance variant explains the proband’s phenotype, clinicians offer testing to parents but not to other family members
32	Impact on patients/Families	How disclosure impacts patients/families	When a low penetrance variant explains the proband’s phenotype, clinicians should offer testing to parents but not to other family members
4	Disclosure decisions	Who decides to disclose	I Consider that parents/probands should have an active role in the decision-making process about the information they want to know
9	Disclosure decisions	Who decides to disclose	Parental preferences should determine which results are disclosed, not the opinions of clinicians
18	Disclosure decisions	Who decides to disclose	National guidelines for variants of uncertain clinical significance may not be applicable to individual cases and each case should be discussed/thought separately
25	Disclosure decisions	Who decides to disclose	Whether to disclose variants of unknown/uncertain clinical significance to parents should be discussed in a national panel of experts
1	X	How disclosure impacts patients/families	When a low penetrance variant explains the proband’s phenotype, I believe clinicians should offer testing to the parents and all family members who wish to be tested
5	X	When to disclose	In our service, all findings, including variants of uncertain significance or low penetrance, are reported only in postnatal cases
10	X	How disclosure impacts fetus/embryos	One reason why information about variants of uncertain clinical significance should not be disclosed to parents is that it may result in the termination of healthy pregnancies
11	X	How disclosure impacts fetus/embryos	I Believe low penetrance CNVs should be investigated in the context of pre-implantation genetic diagnosis when the purpose of the technique is a monogenic disorder
12	X	When to disclose	The laboratory should disclose results of uncertain clinical significance (low penetrance) only in postnatal cases
13	X	When to disclose	In our service, only findings that explain the fetal phenotype are disclosed
17	X	How disclosure impacts fetus/embryos	One reason why variants of uncertain clinical significance should be disclosed is that it gives parents the option of pregnancy termination
26	X	When to disclose	In our service, all findings, including variants of uncertain significance or low penetrance, are disclosed regardless of the context
29	X	When to disclose	The laboratory should not disclose results of clinically uncertain significance (low penetrance), even in prenatal cases
30	X	How disclosure impacts patients/families	In my service, when a low penetrance variant explains the proband’s phenotype, clinicians offer testing to parents and all family members who wish to be tested

First, a strong correlation between Q23 and Q30 (τ = - 0.575, p = 0.001), Q15 and Q31 (τ = - 0.642, p < 0.001), Q15 and Q27 (τ = 0.663, p < 0.001), Q12 and Q29 (τ = 0.643, p < 0.001) and Q3 and Q19 (τ = 0.637, p = 0.002), as well as a moderate correlation between Q27 and Q31 (τ = - 0.469, p = 0.008), allowed us to validate our results. Each pair of stated correlations were used to assess the same outcome but were formulated slightly differently or in reverse (regarding negative correlations). These correlations show that each Clinical Geneticist was consistent in their responses to the same topic throughout the survey. Though the limited sample prevented other more appropriate statistical tests, it was possible to conclude these correlations as approximate reliability metrics for the questionnaire. Table 5 represents a summary descriptive statistics of the responses to all 32 survey questions.

**TABLE 5:** Descriptive statistics of the survey responses, represented by their median, interquartile range (IQR), minimum (Min.) and maximum (Max.) values.

Question	Median	IQR	Min	Max	Question	Median	IQR	Min	Max
Q1	4,00	1,00	2	5	Q17	2,00	1,00	1	4
Q2	2,50	2,00	1	5	Q18	3,50	1,75	1	5
Q3	4,00	1,00	3	5	Q19	4,00	1,00	4	5
Q4	4,00	1,00	3	5	Q20	4,00	1,00	3	5
Q5	3,50	2,00	1	5	Q21	2,00	2,00	1	4
Q6	4,00	1,00	3	5	Q22	3,00	1,00	1	4
Q7	3,00	2,00	1	5	Q23	2,00	1,00	1	5
Q8	5,00	1,00	2	5	Q24	3,00	1,00	1	5
Q9	3,00	1,75	1	5	Q25	4,00	1,75	2	5
Q10	2,00	2,00	1	5	Q26	4,00	2,00	1	5
Q11	2,00	2,00	1	5	Q27	3,00	2,00	1	5
Q12	3,00	2,00	1	5	Q28	2,00	2,00	1	5
Q13	2,00	2,00	1	5	Q29	2,00	2,00	1	5
Q14	4,00	0,00	3	5	Q30	4,00	1,00	2	5
Q15	3,00	2,00	1	5	Q31	3,00	1,75	1	5
Q16	3,00	2,00	1	5	Q32	2,00	1,00	1	4

### 3.5 Disclosure decisions

The strong correlation between Q18 and Q25 (τ = - 0.603, p = 0.001) shows that those who think that decisions on reporting should be discussed in a panel of experts do not agree that each case should be thought of individually, but rather through guideline recommendations. The moderate correlations between Q4 and Q25 (τ = - 0.383, p = 0.039), Q2 and Q25 (τ = 0.485, p = 0.006) and between Q9 and Q25 (τ = - 0.456, p = 0.010) also show that the responders consider that the lab should report all results only in postnatal setting, and do not consider that parents should determine, or have a role deciding on, which LP-CNVs should be reported.

On the other hand, the moderate correlations between Q2 and Q18 (τ = - 0.353, p = 0.047), Q6 and Q18 (τ = - 0.393, p = 0.038) and Q9 and Q18 (τ = 0.353, p = 0.047) revealed that those responders who think that the decisions should be personalized, do not agree that labs should report all findings only in postnatal, and think that parents should determine which variants should be reported, and not the clinicians.

Consequently, the ‘uncertainty challenge' is dual-layered: while clinicians struggle to categorize findings, the resulting information provided to parents is inherently filtered through the clinician’s own ethical bias, leaving patients to struggle with integrating these filtered results into reproductive plans.

Furthermore, the management of LP-CNVs places the principle of parental autonomy in direct tension with the ‘Best Interest of the Child' (BIOC) standard. While parents have a right to comprehensive genetic information to inform reproductive or clinical choices, the low penetrance and variable expressivity of these variants mean that disclosure may unnecessarily impose a ‘patient' status on a child who might never develop a clinical phenotype. In the context of LP-CNVs, where the clinical outcome remains highly uncertain, the BIOC standard suggests a cautious approach to disclosure to protect the child from potential over-medicalization, stigmatization, or the compromise of their ‘right to an open future' (Zawati et al., 2014). This protective stance acknowledges that while parental autonomy is a primary pillar of genetic counseling, it is not absolute and must be balanced against the risk of transforming a healthy child into an asymptomatic patient based on incomplete penetrance.

The results of LP-CNV disclosure in this study reveal a profound ethical tension between standardized, expert-driven reporting and the imperative of individualized patient care. The strong negative correlation between the preference for expert panels and personalized management suggests that a move toward standardization may inadvertently undermine the principle of patient autonomy. While Shkedi-Rafid (2016) suggests that healthcare professionals value patient involvement, our data show that clinicians who rely on expert-led guidelines often exclude parents from the decision-making process regarding which variants should be reported. The observed discrepancies in clinician perspectives, particularly regarding parental involvement and the context-dependent reporting of results, underscore a critical lack of consensus (Shkedi-Rafid et al., 2016).

In the absence of a unified professional standard, the observed lack of consensus among clinicians constitutes a significant breach of distributive justice, which concerns the fair division of benefits and burdens in society. Our findings reveal a ‘postcode lottery' within the Portuguese healthcare system, where the disclosure of an LP-CNV depends more on local hospital protocols than on unified national standards. This variability suggests that a patient’s ‘right to know' is contingent upon geographical factors; specifically, patients attending specialized genetic services in the North of Portugal may be subject to different disclosure criteria than those in the South or Central regions, depending on whether their local hospital prioritizes standardized expert-led guidelines or individualized clinical judgment. Such regional disparities mean that two families facing the same genetic finding could receive conflicting reproductive options or clinical advice, simply because of local institutional preferences. This inconsistency not only compromises the principle of equity in healthcare access but also creates a system where the management of genetic uncertainty is dictated by a provider’s location rather than a patient’s clinical need. As Farrelly (2003) argues, this transforms a biological ‘natural lottery' into a systemic inequality that the state has a moral obligation to regulate (Farrelly and Cooper, 2003).

Furthermore, the results indicate that these ethical failures may stem from a lack of context-sensitive frameworks. The tendency to report all results only in postnatal settings suggests that clinicians apply different ethical weights to the same information depending on the clinical environment. As Gunson (2018) notes, justice is often context-dependent; therefore, the Portuguese system’s failure to adapt its institutions to the distinct needs of prenatal versus postnatal care creates a ‘contextual inequity'. This inequity is particularly striking because the objective genomic data—the LP-CNV itself—remains identical across both settings. Nevertheless, the ethical weight and clinical reporting thresholds diverged significantly based solely on the clinical setting the patient occupied, despite the objective genomic data remaining identical (Gunson, 2018).

### 3.6 Impact on patients/families

The strong correlation between Q3 and Q6 (τ = - 0.559, p = 0.005) and Q3 and Q7 (τ = - 0.552, p = 0.004) shows that responders believe that the more patients struggle to understand results, the more anxiety they feel, and physicians should not choose the results to be reported according to “what patients want to know”. Moreover, after observing a strong correlation between Q8 and Q19 (τ = 0.575, p = 0.005), we understand that the responders who think the reported variants should be provided by national guidelines also believe that parents may struggle to understand genetic data. Furthermore, the significant correlation observed between Q8 and Q19 (τ = 0.575, p = 0.005) suggests that respondents who favor national guidelines for variant reporting also acknowledge the potential for parental difficulty in comprehending genetic information.

The strong correlation between Q6 and Q19 (τ = - 0.573, p = 0.001) suggests that patients who struggle more to understand genetic information experience less anxiety about incidental findings. The widespread health illiteracy and reliance and trust on medical professionals for healthcare guidance may explain this. Also, the strong correlation between Q20 and Q21 (τ = - 0.522, p = 0.006) and the moderate correlations between Q19 and Q21 (τ = - 0,392 p = 0.041), and Q14 and Q21 (τ = −0.429, p = 0.020), reveal that those responders who believe that LP-CNVs should be reported, work in a department where LP-CNVs are reported, and believe that patients may have difficulty understanding the information, think that should not be the clinician who receives the report to decide which information should be reported to patients.

Our results highlight ethical concerns about the potential compromise of decision-making capacity when individuals are presented with highly complex information in a vulnerable situation, and the subsequent implications for the adequacy of informed consent. Upholding the principles of beneficence and non-maleficence, to act in the best interest of the patients and avoid harm, alongside autonomy and the protection of vulnerable persons/families to ensure their decision-making capacity in complex scenarios, presents significant ethical dilemmas in reproductive decision-making.

These findings also underscore the imperative of a shared decision-making approach, which, despite the tendency in genetics to favor “nondirectiveness” in genetic counselling, is supported by current research and guidelines and emphasizes the use of patient decision aids and collaborative communication models to ensure patients are active participants in their healthcare decisions.

The Portuguese healthcare system does not currently recognize Genetic Counselors as an autonomous professional category; instead, the law defines genetic counseling as a ‘medical act' that may be performed only by Clinical Geneticists. This model forces physicians to consolidate complex diagnostic interpretation with psychosocial support. Such consolidation may inadvertently favor a more directive approach to disclosure, as the time-intensive nature of non-directive counseling–essential for navigating the uncertainty of LP-CNVs–may conflict with rising clinical and diagnostic demands. Ultimately, this structural limitation may limit opportunities for values-based deliberation and comprehensive patient education, which characterize healthcare systems with a dedicated genetic counseling workforce.

This lack of professional consensus further challenges the sustainability of the traditional non-directive counseling model. In this context, non-directiveness may become a shell for clinical inconsistency, as the choice offered to the patient is provided by the arbitrary perspective of the provider they happen to consult. Our findings suggest that when clinicians themselves disagree on whether an LP-CNV constitutes a result worth reporting, the information provided to parents is inherently filtered through the clinician’s own ethical bias. While SDM aims to empower patients, our results reveal that clinicians often favor a clinician-directed disclosure model, rooted in the perception that families may be overwhelmed by the technical uncertainty of genomic data (Q20, Q21, and Q14). This tension is further exacerbated by the shift toward shared decision-making (SDM) approach (Elwyn et al., 2000). While SDM aims to empower patients, our results reveal that clinicians often favor a clinician-directed disclosure model (Elwyn et al., 2000).

In the same line, the correlations between Q20, Q21, Q19, Q21, and Q14, Q21 reveal a tendency among clinicians to favor clinician-directed information disclosure, based on the perceived difficulty patients have in understanding the information. The significant correlation between Q8 and Q19 underscores the general trend of the results, pointing to the need for a shared decision-making approach, where the clinicians and the patients make the decisions together. This approach, however, necessitates that healthcare professionals receive adequate training and education to ensure effective communication and informed consent (Birch et al., 2018). The potential psychological impact of receiving and interpreting genetic information about LP-CNVs should not be underestimated. Access to appropriate counselling and support services is crucial to address the emotional and psychological needs of individuals and families facing these complex issues.

Interestingly, the strong correlation between Q23 and Q32 (τ = 0.767, p < 0.001) confirms that clinicians working in departments where the study of a previously identified CNV is offered to parents, but not to other family members, endorse this procedure. However, the complexities of genetic information extend beyond the individual patient, often impacting family dynamics, communication, and relationships. Genetic counselling services must be equipped to address these intricate familial issues, offering support and guidance to individuals and families as they navigate the challenges of genetic testing and its potential consequences. The “spillover effect” of genetic testing results within families, where a single genetic variant can have different implications and interpretations for different family members, can lead to conflicts and tensions.

This raises ethical issues about equitable access to genetic information. While a holistic approach is essential for responsible management, the principle of justice–specifically the fair allocation of resources–must be carefully weighed. As discussed by Farrelly (2002), the state and healthcare institutions must balance the individual’s ‘brute luck' in the natural lottery with the public good. Consequently, the equitable allocation of resources must be considered at both the individual level (e.g., testing extended family members) and the societal level (e.g., the rationing of scarce resources in public health systems) (Farrelly and Cooper, 2003).

### 3.7 Reproductive options

Another important finding was a moderate correlation between Q2 and Q28 (τ = - 0.495, p = 0.005), confirming that those who think all results should be reported only in postnatal diagnosis also think that not all the findings should be reported in the prenatal setting. This illustrates that the sensitivity of prenatal situations, which bring more anxiety and may have more dramatic outcomes, differs from the sensitivity in postnatal situations, where any result can be a clue for the answer to the diagnosis of a child with a neurodevelopmental disorder. The idea that the lab should report only variants that explain a fetal phenotype is supported by those responders who work in a department where only these variants are reported in the prenatal setting, as well as those who think that parents should not choose what to disclose (but rather after guidelines). This is suggested by the moderate correlations Q7 and Q16 (τ = - 0.351, p = 0.043), Q8 and Q16 (τ = 0.429, p = 0.018), Q9 and Q16 (τ = - 0.381, p = 0.031), and Q13 and Q16 (τ = 0.356, p = 0.041).

However, the strong correlation between Q16 and Q28 (τ = - 0.524, p = 0.003) shows that those who think the lab should report disclose only variants that provide a clinical explanation for the found fetal anomaly, do not agree that lab should disclose all findings, regardless of their clinical significance, even in prenatal cases. These results may suggest a tendency for clinicians to prioritize reporting variants that “explain the fetal phenotype”, effectively limiting information based on perceived clinical relevance, potentially undermining parental autonomy. Ethically, this also raises questions about the transparency of information disclosure and the potential for clinicians to impose their values on reproductive decisions.

On the other hand, the strong correlation between Q7 and Q15 (τ = - 0.573, p < 0.001) and Q7 and Q27 (τ = - 0.512, p = 0.003), as well as the moderate correlation Q7 and Q24 (τ = - 0.489, p = 0.005) revealed that the more responders think that the physician should choose which LP-CNVs should be reported or not, the less they think PND/PGT should be offered. This suggests that, if physicians determine which LP-CNVs to report, based primarily on clinical presentation (i.e., those that explain the phenotype), they would limit reporting to variants deemed actionable, such as those eligible for PND/PGT. These results indicate that clinicians who favor controlled variant reporting are less likely to support offering PND/PGT. This suggests a potential bias, where clinicians may limit access to reproductive options based on their interpretations of LP-CNV significance. Thus, ethical concerns regarding reproductive autonomy and the potential for discrimination based on genetic information are raised.

The moderate correlation between Q15 and Q24 (τ = 0.388, p = 0.030) indicates that respondents who support offering PND for LP-CNVs also work in departments providing PGT. While this aligns with reproductive options, further in-depth ethical analysis of these practices is warranted.

Another important and ethically questionable result is the strong correlation between Q2 and Q27 (τ = 0.561, p = 0.002), which reveals that those responders in favor of PND are also in favor of embryos with an LP-CNV being “excluded” from potential pregnancy. Also, the strong correlation between Q22 and Q31 (τ = - 0.510, p = 0.005) confirms consistency, as responders who think that embryos carrying an LP-CNV should be excluded also believe that PGT should be offered for this reason. These correlations reveal a link between supporting PND and advocating for the “exclusion” of embryos with LP-CNVs, a trend that evokes the historical misuse of genetic information and underscores the importance of guarding against modern eugenic practices (Lombado, 2003). The low penetrance for mild neurodevelopmental issues make these variants questionable for PGT as the most individuals carrying these CNVs are healthy (in contrast to other disorders currently considered acceptable for PGT). If these were to be considered susceptibility variants for neurodevelopmental disorders, we wonder if it would be morally acceptable to offer PGT for them while not offering PGT for susceptibility variants associated with other multifactorial disorders, such as diabetes or autoimmune diseases.

Our results also highlight the need for careful consideration of the potential for stigmatization and discrimination against individuals with LP-CNVs. This potential for genetic ‘labeling' adds a layer of ethical complexity to reproductive choices, as the decision to select against these variants raises profound moral and societal questions about what constitutes a healthy life (Baynam et al., 2024). Ultimately, the decision to select against embryos with LP-CNVs challenges our definitions of health and disease, shifting the focus from clinical necessity to a subjective determination of a ‘life worth living' – a shift that requires urgent national consensus to protect both parental autonomy and the rights of the future child.

## 4 Conclusive notes

Our findings confirm daily challenges faced by Clinical Genetics in Portugal, particularly concerning the ethical complexities surrounding LP-CNVs. While the sample is small (n = 24), it represents over 50% of the active specialists in the country, justifying the exploratory nature of the study. Even so, further studies may involve other European countries through collaborations, since this is a common challenge for clinical Genetics. Although this study addresses a previously identified issue, and countries such as the United Kingdom and Belgium have established guidelines on LP-CNVs’ disclosure and impact on clinical conduct and reproductive options, other countries, such as Portugal do not have any national guidance governing the interpretation or clinical management of LP-CNVs. As so, this study was important to confirm and streamline the situation and the real problems in a country with growing genetic care expertise, allowing for the reinforcement of the ethical dilemmas that can and should be raised and considered by the lack of standardized national guidelines that leads to inconsistent practices, violating the principle of justice and creating a “postcode lottery” for patients. This absence of consensus also impacts reproductive decision-making, raising ethical dilemmas concerning beneficence, non-maleficence, and autonomy, particularly regarding prenatal diagnosis and preimplantation genetic testing, and the potential for genetic discrimination. Effective communication, informed consent, and shared decision-making are crucial, requiring better training for healthcare professionals and access to robust counselling services to address the psychological and familial impact of LP-CNV information. Ultimately, the findings underscore the urgent need for a concerted, multidisciplinary effort to establish a robust ethical framework that elucidates the uncertainties surrounding LP-CNVs, upholds patient autonomy, guarantees equitable access, and protects against potential genetic discrimination.

This framework must emphasize the development of comprehensive, standardized guidelines, cultivate a culture of shared decision-making, and invest in the education and training of healthcare professionals. Ultimately, by adopting a holistic approach that acknowledges the intricate interplay of individual, familial, and societal factors, we can navigate the evolving landscape of clinical genetics with wisdom, compassion, and unwavering ethical integrity, ensuring that the potential of genetic knowledge empowers rather than marginalizes the individuals and families it aims to assist.

## Data Availability

The original contributions presented in the study are included in the article/Supplementary Material, further inquiries can be directed to the corresponding author.

## References

[B1] Baynam G. Gomez R. Jain R. (2024). Stigma associated with genetic testing for rare Diseases—causes and recommendations. Front. Genet. 15, 1335768. 10.3389/fgene.2024.1335768 38638122 PMC11024281

[B2] BeSHG (2024). Prenatal array guidelines. Available online at: https://www.college-genetics.be/assets/recommendations/fr/guidelines/BeSHG%20prenatal%20consortium_guidelines%20prenatal%20array_V2023.pdf?utm_source=chatgpt.com.

[B3] Birch P. H. Adam S. Coe R. R. Port A. V. Vortel M. Friedman J. M. (2018). Assessing shared decision-making clinical behaviors among genetic counsellors. J. Genet. Couns. 28, 40–49. 10.1007/s10897-018-0285-x 30109450

[B4] Christiansen J. Dyck J. D. Elyas B. G. Lilley M. Bamforth J. S. Hicks M. (2004). Chromosome 1q21.1 contiguous gene deletion is associated with congenital heart disease. Circulation Res. 94 (11), 1429–1435. 10.1161/01.RES.0000130528.72330.5c 15117819

[B5] Cooper G. M. Coe B. P. Girirajan S. Rosenfeld J. A. Vu T. H. Baker C. (2011). A copy number variation morbidity map of developmental delay. Nat. Genet. 43 (9), 838–846. 10.1038/ng.909 21841781 PMC3171215

[B6] Elwyn G. Gray J. Clarke A. (2000). Shared decision making and non-directiveness in genetic counselling. J. Med. Genet. 37 (2), 135–138. 10.1136/jmg.37.2.135 10662816 PMC1734519

[B7] Farrelly C. (2003). “Distributive justice and genetics,” in Nature encyclopedia of the human genome. Editor Cooper D. N. (London: Nature Publishing Group), 2, 42–44.

[B8] Gardiner C. (2015). Recommendations for the use of chromosome microarray in pregnancy. Available online at: https://www.rcpath.org/static/06664c28-0f90-4230-86158c91fea14be6/818ebc78-3182-410f-870685879f92d242/Recommendations-for-the-use-of-chromosome-microarray-in-pregnancy.pdf.

[B9] Goh S. Thiyagarajan L. Dudding-Byth T. Pinese M. Kirk E. P. (2025). A systematic review and pooled analysis of penetrance estimates of copy-number variants associated with neurodevelopment. Genet. Med. 27 (1), 101227. 10.1016/j.gim.2024.101227 39092588

[B10] Govaerts L. Srebniak M. Diderich K. Joosten M. Riedijk S. Knapen M. (2017). Prenatal diagnosis of susceptibility loci for neurodevelopmental disorders - genetic counseling and pregnancy outcome in 57 cases: prenatal diagnosis of susceptibility CNVs. Prenat. Diagn. 37 (1), 73–80. 10.1002/pd.4979 27931090

[B11] Gunson D. (2018). Genetics and justice: must one theory fit all contexts? Camb Q. Healthc. Ethics 27 (2), 250–260. 10.1017/S0963180117000585 29509117

[B12] Kendall K. M. Bracher-Smith M. Fitzpatrick H. Lynham A. Rees E. Escott-Price V. (2019). Cognitive performance and functional outcomes of carriers of pathogenic copy number variants: analysis of the UK biobank. Br. J. Psychiatry 214 (5), 297–304. 10.1192/bjp.2018.301 30767844 PMC6520248

[B13] Lombado P. (2003). Taking eugenics seriously: three generations of ??? Are enough? Available online at: https://ir.law.fsu.edu/lr/vol30/iss2/2/.

[B14] MacCallum R. C. Widaman K. F. Zhang S. Hong S. (1999). Sample size in factor analysis. Psychol. Methods 4 (1), 84–99. 10.1037/1082-989x.4.1.84

[B15] Miller D. T. Adam M. P. Aradhya S. Biesecker L. G. Brothman A. R. Carter N. P. (2010). Consensus statement: chromosomal microarray is a first-tier clinical diagnostic test for individuals with developmental disabilities or congenital anomalies. Am. J. Hum. Genet. 86 (5), 749–764. 10.1016/j.ajhg.2010.04.006 20466091 PMC2869000

[B16] Morris E. O’Donovan M. Virani A. Austin J. (2021). An ethical analysis of divergent clinical approaches to the application of genetic testing for autism and schizophrenia. Hum. Genet. 141, 1069–1084. 10.1007/s00439-021-02349-1 34453583

[B17] Muys J. Blaumeiser B. Janssens K. Loobuyck P. Jacquemyn Y. (2020). Chromosomal microarray analysis in prenatal diagnosis: ethical considerations of the Belgian approach. J. Med. Ethics 46 (2), 104–109. 10.1136/medethics-2018-105186 31527144

[B18] National Library of Medicine (2020). Proceedings of the 23rd annual meeting of the Portuguese society of human genetics. Medicine 99 (9):e19291.

[B19] Rosenfeld J. A. Coe B. P. Eichler E. E. Cuckle H. Shaffer L. G. (2013). Estimates of penetrance for recurrent pathogenic copy-number variations. Genet. Med. 15 (6), 478–481. 10.1038/gim.2012.164 23258348 PMC3664238

[B20] Shkedi-Rafid S. Fenwick A. Dheensa S. Wellesley D. Lucassen A. M. (2016). What results to disclose, when, and who decides? Healthcare professionals’ views on prenatal chromosomal microarray analysis: healthcare professionals’ views on prenatal CMA testing. Prenat. Diagn. 36 (3), 252–259. 10.1002/pd.4772 26743561 PMC5067646

[B21] Zawati M. H. Parry D. Knoppers B. M. (2014). The best interests of the child and the return of results in genetic research: international comparative perspectives. BMC Med. Ethics 15 (1), 72. 10.1186/1472-6939-15-72 25280986 PMC4192737

