# Supplementary material for: Ethical and clinical challenges in managing low-penetrance CNVs: insights from Portuguese clinical geneticists
Source: Front Genet. 2026 Mar 2;17:1757167. doi: 10.3389/fgene.2026.1757167 (PMC12989213; doi:10.3389/fgene.2026.1757167)
Supplement: Supplementary file 1 [file DataSheet1.pdf]

QUESTIONNAIRE

PART 1

1) Years of Experience as a specialist in Clinical Genetics:
a. > 5
b. > 5

2) In which region of the country is located the public hospital where you work?
a. North Zone
b. Central Zone
c. South Zone

3) In cases of CNVs associated with neurodevelopmental disorder, in my service, Clinical Geneticist (check all that apply):
a. Discusses cases with the Maternal-Fetal Medicine team
b. Refers cases to the Pediatric Neurodevelopment and/or Child Psychiatry consultation
c. Refers cases to General and Family Medicine
d. Reevaluates cases near adulthood and/or during reproductive planning
e. Reevaluates cases when requested

4) What techniques have been used for CNV detection (in the last 10 years)? Please check ALL applicable options:
a. aCGH
b. SNP-array
c. MLPA
d. Other: ______________

5) Where is the cytogenetic study for CNV detection performed?
a. Laboratory within the center
b. NHS-contracted laboratory
c. Privately contracted laboratory

6) If you answered "Contracted Laboratory" in the previous question (NHS or Private), please specify which one. ________________________

7) Regarding the classification used (by the clinician and/or the laboratory you work with) for classifying CNVs in postnatal cases, please check ALL that apply:
a. Pathogenic / Probably pathogenic
b. Variants of unknown significance
c. Susceptibility variants
d. Variants of uncertain significance
e. Variants of uncertain or unknown significance (not differentiated in classification)
f. Benign / Probably benign
g. Other classification

8) Regarding the classification used (by the clinician and/or the laboratory you work with) for classifying CNVs in prenatal cases, please check ALL that apply:
a. Pathogenic / Probably pathogenic
b. Variants of unknown significance
c. Susceptibility variants
d. Variants of uncertain significance
e. Variants of uncertain or unknown significance (not differentiated in classification)
f. Benign / Probably benign
g. Other classification

PART 2

The following questions will be about low penetrance CNVs / susceptibility CNVs for neurodevelopmental pathology. Considering 1- strongly disagree, 2- disagree, 3- neither agree nor disagree or have no opinion, 4- agree, 5- strongly agree, please mark the questions below:

1) When a low penetrance variant explains the proband's phenotype, I believe clinicians should offer testing to the parents and all family members who wish to be tested.
1 2 3 4 5

2) The laboratory should report all findings, regardless of their clinical significance, only in postnatal cases.
1 2 3 4 5

3) I consider that parents/probands may have difficulty understanding the information provided in genetic counseling, especially the possible findings of the genetic study.
1 2 3 4 5

4) I consider that parents/probands should have an active role in the decision-making process about the information they want to know.
1 2 3 4 5

5) In our service, all findings, including variants of uncertain significance or low penetrance, are reported only in postnatal cases.
1 2 3 4 5

6) Disclosure incidental findings can lead to parental anxiety.
1 2 3 4 5

7) I believe the clinician should "choose" the findings to disclosure based on what the parents want to know.
1 2 3 4 5

8) The decision on which information to disclose, especially regarding variants of uncertain clinical significance, should be determined by national guidelines and not left to individual laboratories/clinicians.
1 2 3 4 5

9) Parental preferences should determine which results are disclosure, not the opinions of clinicians.
1 2 3 4 5

10) One reason why information about variants of uncertain clinical significance should not be disclosed to parents is that it may result in the termination of healthy pregnancies.
1 2 3 4 5

11) I believe low penetrance CNVs should be investigated in the context of pre-implantation genetic diagnosis when the purpose of the technique is a monogenic disorder.
1 2 3 4 5

12) The laboratory should disclose results of uncertain clinical significance (low penetrance) only in postnatal cases.
1 2 3 4 5

13) In our service, only findings that explain the fetal phenotype are disclosure.
1 2 3 4 5

14) In my opinion, variants of uncertain clinical significance/low penetrance should be reported.
1 2 3 4 5

15) When a low penetrance variant explains the proband's phenotype, clinicians should offer invasive prenatal diagnosis in future pregnancies.
1 2 3 4 5

16) The laboratory should disclose only variants that provide a clinical explanation for the found fetal anomaly.
1 2 3 4 5

17) One reason why variants of uncertain clinical significance should be disclosed is that it gives parents the option of pregnancy termination.
1 2 3 4 5

18) National guidelines for variants of uncertain clinical significance may not be applicable to individual cases and each case should be discussed/thought separately.
1 2 3 4 5

19) I consider that parents/probands may have difficulties understanding the information provided in genetic counseling.
1 2 3 4 5

20) In my service, variants of uncertain clinical significance/low penetrance are disclosure.
1 2 3 4 5

21) I believe the clinician receiving the report should decide what information to give to parents/probands.
1 2 3 4 5

22) Embryos carrying low penetrance CNVs should be "excluded".
1 2 3 4 5

23) In my service, when a low penetrance variant explains the proband's phenotype, clinicians offer testing to parents but not to other family members.
1 2 3 4 5

24) In my service, when a low penetrance variant explains the proband's phenotype, clinicians offer pre-implantation genetic diagnosis.
1 2 3 4 5

25) Whether to disclosure variants of unknown/uncertain clinical significance to parents should be discussed in a national panel of experts.
1 2 3 4 5

26) In our service, all findings, including variants of uncertain significance or low penetrance, are disclosure regardless of the context.
1 2 3 4 5

27) In my service, when a low penetrance variant explains the proband's phenotype, clinicians offer invasive prenatal diagnosis in future pregnancies.
1 2 3 4 5

28) The laboratory should disclose all findings, regardless of their clinical significance, even in prenatal cases.
1 2 3 4 5

29) The laboratory should not disclose results of clinically uncertain significance (low penetrance), even in prenatal cases.
1 2 3 4 5

30) In my service, when a low penetrance variant explains the proband's phenotype, clinicians offer testing to parents and all family members who wish to be tested.
1 2 3 4 5

31) When a low penetrance variant explains the proband's phenotype, clinicians should not offer pre-implantation genetic diagnosis.
1 2 3 4 5

32) When a low penetrance variant explains the proband's phenotype, clinicians should offer testing to parents but not to other family members.
1 2 3 4 5

(Some questions adapted from the questionnaire in the article Shkedi-Rafid, S., 2016)
